# Supplementary material for: Gemcitabine and ATR inhibitors synergize to kill PDAC cells by blocking DNA damage response
Source: Mol Syst Biol. 2025 Jan 21;21(3):231–53. doi: 10.1038/s44320-025-00085-6 (PMC11876601; doi:10.1038/s44320-025-00085-6)
Supplement: Supplementary file 1 — Appendix [file 44320_2025_85_MOESM1_ESM.pdf]

## Appendix for

# Gemcitabine and ATR inhibitors synergize to kill PDAC cells by blocking DNA damage response

Stefanie Höfer[1], Larissa Frasch[1], Sarah Brajkovic[1], Kerstin Putzker[2], Joe Lewis[2], Hendrik Schürmann[3,4,5], Valentina Leone[6], Amirhossein Sakhteman[1], Matthew The[1], Florian P. Bayer[1], Julian Müller[1], Firas Hamood[1], Jens T. Siveke[3,4], Maximilian Reichert[6,7], and Bernhard Kuster[1,7*]

[1]Chair of Proteomics and Bioanalytics, Technical University of Munich, Freising, Germany

[2]Chemical Biology Core Facility, EMBL Heidelberg, Heidelberg, Germany

[3]Bridge Institute of Experimental Tumor Therapy (BIT) and Division of Solid Tumor Translational Oncology (DKTK), West German Cancer Center, University Hospital Essen, University of Duisburg-Essen, Essen, Germany

[4]German Cancer Consortium (DKTK), partner site Essen, a partnership between German Cancer Research Center (DKFZ) and University Hospital Essen, Germany

[5]Department of Medical Oncology, West German Cancer Center, University Hospital Essen, Essen, Germany

[6]Department of Internal Medicine II, University Hospital rechts der Isar, Technical University Munich, Munich, Germany

[7]German Cancer Consortium (DKTK), Partner Site Munich, Munich, Germany

*Corresponding author. Email: kuster@tum.de

# Table of contents


| Name | Description | Page |
|---|---|---|
| Appendix Figure S1 | Substrates of ATM are not affected in phosphorylation upon ATRi | 1 |
| Appendix Figure S2 | Clinical data implies increased DNA damage response activity in PDAC cells | 2 |
| Appendix Figure S3 | GEM and ATRi affect expression of nucleotide metabolism enzymes over time | 3 |
| Appendix Table S1 | Information on 13 PDAC cell lines used for drug screening | 4 |
| Appendix Table S2 | Information on nine pancreatic PDOs used to verify GEM+ATRi synergy | 5 |
| Appendix Table S3 | Information on reagents and buffers used for experiments with PDOs | 6 |
| Appendix Table S4 | TMT-mapping for phospho-experiments in AsPC-1 cells (decryptM) | 7 |
| Appendix Table S5 | TMT-mapping for phospho-experiments in six other PDAC cell lines (non-decryptM) | 8 |
| Appendix Table S6 | TMT-mapping for time-dependent full proteome experiment in AsPC-1 cells | 9 |
| References | References used for Appendix | 10 |

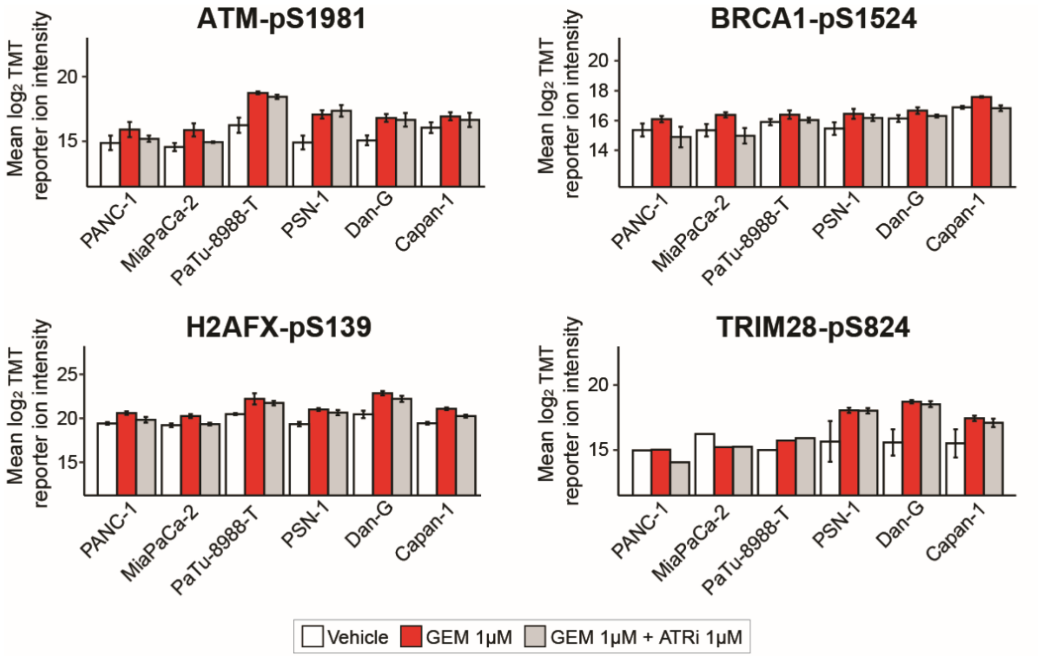


**Appendix Figure S1. Substrates of ATM are not affected in phosphorylation upon ATRi.**
TMT reporter ion intensity ($log_2$) of selected ATM substrates in six PDAC cell lines treated with vehicle, 1 µM GEM, or 1 µM GEM plus 1 µM Elimusertib. Data are presented as mean values, with error bars representing the ± s.d. of quadruplicates (n = 4). Information on kinase-substrate-relationship based on *PhosphoSitePlus* (Hornbeck *et al*, 2015).

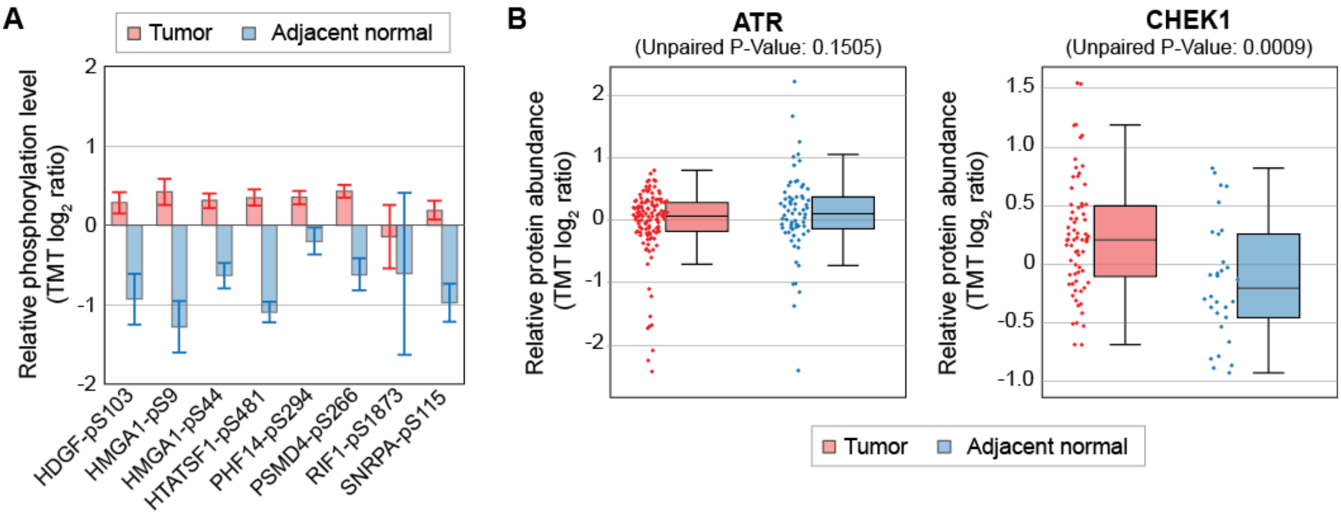


**Appendix Figure S2. Clinical data implies increased DNA damage response activity in PDAC cells.**

**A.** Relative phosphorylation levels of 8 of the 36 pSQ/pTQ sites in PDAC tumor (red) vs. adjacent normal cells (blue). The counts of tumor and adjacent normal cells are: 38 and 19 (HDGF-pS103), 11 and 5 (HMGA1-pS9), 85 and 40 (HMGA1-pS44), 46 and 25 (HTATSF1-pS481), 15 and 8 (PHF14-pS294), 26 and 11 (PSMD4-pS266), 5 and 2 (RIF1-pS1873, or 36 and 16 (SNRPA-pS115), respectively. Data are presented as mean values, and error bars indicate the standard error.

**B.** Relative protein abundance of ATR and CHEK1 in PDAC tumor (red) vs. adjacent normal cells (blue). Unpaired p-values were calculated using the Mann-Whitney U-test. Tumor counts: 124 (ATR) and 69 (CHEK1); adjacent normal cells counts: 59 (ATR) and 30 (CHEK1). The boxplots display the median (center), the interquartile range (bounds of the box), and the upper and lower fences (whiskers) of relative protein abundances.

Data information: The data were generated by the National Cancer Institute Clinical Proteomic Tumor Analysis Consortium (CPTAC) and accessed through Proteomic Data Commons (Thangudu *et al*, 2020). The analysis and plots were generated using the embedded web tool *cProSite* (https://cprosite.ccr.cancer.gov/) (Wang *et al*, 2023).

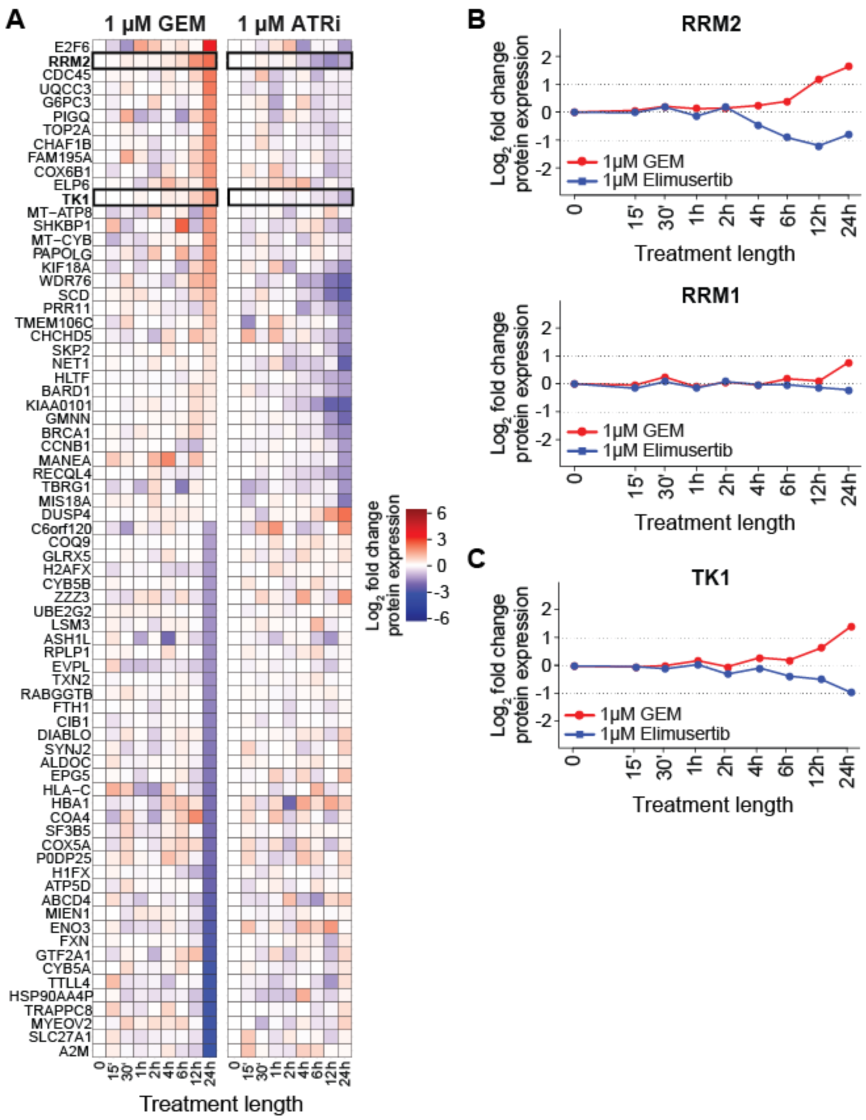


**Appendix Figure S3. GEM and ATRi affect expression of nucleotide metabolism enzymes over time. A.** $Log_2$ fold changes in protein expression in AsPC-1 cells treated with 1 µM GEM or 1 µM Elimusertib over time relative to untreated cells. **B-C.** $Log_2$ fold changes in protein expression over time upon treatment with 1 µM GEM (red) or 1 µM Elimusertib (blue) of RRM2 (B, upper), RRM1 (B, lower), and TK1 (C).

| Cell line | Supplier or provider | Supplier order # | Medium | Medium supplements** | Cell density (384-well) |
|---|---|---|---|---|---|
| AsPC-1 | ATCC | CRL-1682 | DMEM | 10% FBS, 1% non-essential amino acids | 1000 |
| PANC-1 | ATCC | CRL-1469 | DMEM | 10% FBS | 2000 |
| PSN-1 | ATCC | CRL-3211 | RPMI | 10% FBS | 1000 |
| BxPC-3 | ATCC | CRL-1687 | RPMI | 10% FBS | 1000 |
| MiaPaCa-2 | ATCC | CRL-1420 | DMEM | 10% FBS | 1000 |
| Capan-1 | CLS | 300143 | RPMI | 10% FBS, 1% HEPES | 2000 |
| FAMPAC | CLS | 300309 | RPMI | 10% FBS, 1% HEPES | 1000 |
| HuP-T4 | creative-bioarray | CSC-C0333 | DMEM/Hams-F12 (1:1) | 10% FBS, 15 mM HEPES | 1000 |
| Dan-G | DSMZ | ACC-249 | RPMI | 10% FBS | 1000 |
| PaTu-8988-T | DSMZ | ACC-162 | DMEM | 10% FBS | 1000 |
| Colo-357 | Molecular Oncology Department of Radiation, LMU* | - | DMEM | 10% FBS | 2000 |
| HPDE | Molecular Oncology Department of Radiation, LMU* | - | RPMI/keratinocyte serum-free medium (1:1) | 10% FBS, 5 ng/ml human epidermal growth factor, 50 µg/ml bovine pituitary extract | 1000 |
| Suit2-07 | Molecular Oncology Department of Radiation, LMU* | - | RPMI | 10% FBS | 1000 |

*Cell lines received from Molecular Oncology Department of Radiation, LMU were kindly provided by Prof Kisten Lauber

**For high-throughput drug screening, all cell culture medium was supplemented with 1% 100 U/mL penicillin and 100 ug/mL streptomycin.

**Appendix Table S1. Information on 13 PDAC cell lines used for drug screening.** Information on supplier and order number (if applicable) of screened cell lines is given, as well as cell culture medium, supplements and cell densities seeded per well of a 384-well plate.

| PDO set | PDO | Age at diagnosis/ operation | Sex | Site | Sample | KRAS Mutation |
|---|---|---|---|---|---|---|
| 1 | B250 | 71 | male | pancreas | surgical | G12D |
| 1 | B535 | 44 | male | pancreas | surgical | G12D |
| 1 | B678 | 54 | male | pancreas | endosonography | G12V |
| 2 | UME008 | 57 | male | pancreas | surgical | G12D |
| 2 | UME012 | 65 | male | ascites | ascites | G12D |
| 2 | UME051 | 53 | male | pancreas | endosonography | G12D |
| 2 | UME053 | 56 | female | metastasis (abdominal wall) | surgical | WT |
| 2 | UME060 | 65 | male | metastasis (liver) | endosonography | G12V |
| 2 | UME061 | 70 | female | ascites | ascites | G12R |

**Appendix Table S2. Information on nine pancreatic PDOs tested in viability assays.** Information on the age and sex of the donor is given, as well the site and procedure of retrieval, and the KRAS mutational state.

| Samples | Medium name | Component | Final concentration |
|---|---|---|---|
| PDO set 1 | Anti-Anti solution | PBS | 1x |
| PDO set 1 | Anti-Anti solution | Anti-Anti (Merck, A5955) | 1x |
| PDO set 1 | Anti-Anti solution | Gentamicin (Merck, G1272) | 100μg/mL |
| PDO set 1 | Washing medium | Advanced DMEM/F12 (Thermo Fisher , 12634-010) | 1X |
| PDO set 1 | Washing medium | Glutamax (Thermo Fisher, 35050038) | 1X |
| PDO set 1 | Washing medium | HEPES (Thermo Fisher, 15630056) | 1X |
| PDO set 1 | Washing medium | Primocin (InvivoGen) | 1X |
| PDO set 1 | Digestion buffer | Washing medium | - |
| PDO set 1 | Digestion buffer | Collagenase Type II (Thermo Fisher, 17101015) | 6 mg/ml |
| PDO set 1 | Digestion buffer | Dispase II (Merck, 4942078001) | 100 μg/ml |
| PDO set 1 | PDAC PDO medium | Advanced DMEM/F12 (Thermo Fisher, 12634-010) | 1X |
| PDO set 1 | PDAC PDO medium | Glutamax (Thermo Fisher, 35050038) | 1X |
| PDO set 1 | PDAC PDO medium | HEPES (Thermo Fisher, 15630056) | 1X |
| PDO set 1 | PDAC PDO medium | Penicillin/Streptomicin (Thermo Fisher, 15140122) | 1% |
| PDO set 1 | PDAC PDO medium | R-spondin conditioned medium (home made) | 10% (v/v) |
| PDO set 1 | PDAC PDO medium | B-27 Supplement | 1X |
| PDO set 1 | PDAC PDO medium | N-2 Supplement | 1X |
| PDO set 1 | PDAC PDO medium | N-acetylcysteine | 1 mM |
| PDO set 1 | PDAC PDO medium | Recombinant human EGF | 50 ng/ml |
| PDO set 1 | PDAC PDO medium | A83-01 | 0.5 μM |
| PDO set 1 | PDAC PDO medium | Primocin | 100 μg/ml |
| PDO set 1 | PDAC PDO medium | Recombinant human Noggin | 25 ng/ml |
| PDO set 1 | PDAC PDO medium | Recombinant human Wnt3A | 50 ng/ml |
| PDO set 1 | PDAC PDO medium | Nicotinamide | 10 mM |
| PDO set 1 | PDAC PDO medium | Recombinant human FGF-10 | 100 ng/ml |
| PDO set 1 | PDAC PDO medium | human Gastrin-I | 1 nM |
| PDO set 1 | PDAC PDO medium | Prostaglandin-2 | 1 μM |
| PDO set 2 | Anti-Anti solution | Washing medium | - |
| PDO set 2 | Anti-Anti solution | Anti-Anti (Thermo Fisher, 15240-062 ) | 1x |
| PDO set 2 | Washing medium | Advanced DMEM/F12 (Gibco) | 1X |
| PDO set 2 | Washing medium | GlutaMAX (Gibco, 35050061) | 1X |
| PDO set 2 | Washing medium | HEPES (Gibco, 15-630-080) | 1X |
| PDO set 2 | Washing medium | Primocin (InvivoGen) | 125 μg/ml |
| PDO set 2 | Digestion buffer | Washing medium | - |
| PDO set 2 | Digestion buffer | Collagenase Type II (Thermo Fisher, 17101015) | 5 mg/ml |
| PDO set 2 | Digestion buffer | Dispase II (Life Technologies, 17105041) | 1.25 mg/ml |
| PDO set 2 | Digestion buffer | Trypsin inhibitor (Thermo Fisher, 17075029) | 1 mg/ml |
| PDO set 2 | PDAC PDO medium | Advanced DMEM/F12 (Gibco) | 1X |
| PDO set 2 | PDAC PDO medium | GlutaMAX (Gibco, 35050061) | 1X |
| PDO set 2 | PDAC PDO medium | HEPES (Gibco, 15-630-080) | 1X |
| PDO set 2 | PDAC PDO medium | R-spondin conditioned medium (home made) | 10% (v/v) |
| PDO set 2 | PDAC PDO medium | B-27 Supplement | 1X |
| PDO set 2 | PDAC PDO medium | N-acetylcysteine | 1 mM |
| PDO set 2 | PDAC PDO medium | Recombinant human EGF | 50 ng/ml |
| PDO set 2 | PDAC PDO medium | A83-01 | 0.5 μM |
| PDO set 2 | PDAC PDO medium | Primocin | 125 μg/mL |
| PDO set 2 | PDAC PDO medium | Recombinant human Noggin | 100 ng/ml |
| PDO set 2 | PDAC PDO medium | Wnt3a-conditioned medium (home made) | 50% (v/v) |
| PDO set 2 | PDAC PDO medium | Nicotinamide | 10 mM |
| PDO set 2 | PDAC PDO medium | Recombinant human FGF-10 | 100 ng/ml |
| PDO set 2 | PDAC PDO medium | Human Gastrin-I | 10 nM |

**Appendix Table S3. Information on media and buffers used handling pancreatic PDOs.** PDO set 1: B250, B535, B678; PDO set 2: UME008, UME012, UME051, UME053, UME060, UME061.

| TMT Channel | Cell line | GEM [nM] | ATRi [nM] |
|---|---|---|---|
| TMT11: 11-plex 131H | AsPC-1 | 0 | 0 |
| TMT10: 10-plex 131L | AsPC-1 | 1000 | 0 |
| TMT10: 10-plex 130H | AsPC-1 | 1000 | 1 |
| TMT10: 10-plex 130L | AsPC-1 | 1000 | 3 |
| TMT10: 10-plex 129H | AsPC-1 | 1000 | 10 |
| TMT10: 10-plex 129L | AsPC-1 | 1000 | 30 |
| TMT10: 10-plex 128H | AsPC-1 | 1000 | 100 |
| TMT10: 10-plex 128L | AsPC-1 | 1000 | 300 |
| TMT10: 10-plex 127H | AsPC-1 | 1000 | 1000 |
| TMT10: 10-plex 127L | AsPC-1 | 1000 | 3000 |
| TMT10: 10-plex 126 | AsPC-1 | 1000 | 10000 |

**Appendix Table S4. TMT-mapping for phospho-experiments in AsPC-1 cells (decrypM).** The tested ATRi were Elimusertib, Gartisertib, Berzosertib, and Ceralasertib.

| | TMT Channel | Cell line | GEM [nM] | Elimusertib [nM] |
|---|---|---|---|---|
| TMT batch 1 | TMT10: 10-plex 131L | AsPC-1 | 1000 | 1000 |
| | TMT10: 10-plex 130H | Panc-1 | 1000 | 1000 |
| | TMT10: 10-plex 130L | Panc-1 | 1000 | 0 |
| | TMT10: 10-plex 129H | Panc-1 | 0 | 0 |
| | TMT10: 10-plex 129L | PaTu-8988-T | 1000 | 1000 |
| | TMT10: 10-plex 128H | PaTu-8988-T | 1000 | 0 |
| | TMT10: 10-plex 128L | PaTu-8988-T | 0 | 0 |
| | TMT10: 10-plex 127H | MiaPaCa-2 | 1000 | 1000 |
| | TMT10: 10-plex 127L | MiaPaCa-2 | 1000 | 0 |
| | TMT10: 10-plex 126 | MiaPaCa-2 | 0 | 0 |
| TMT batch 2 | TMT10: 10-plex 131L | AsPC-1 | 1000 | 1000 |
| | TMT10: 10-plex 130H | Dan-G | 1000 | 1000 |
| | TMT10: 10-plex 130L | Dan-G | 1000 | 0 |
| | TMT10: 10-plex 129H | Dan-G | 0 | 0 |
| | TMT10: 10-plex 129L | PSN-1 | 1000 | 1000 |
| | TMT10: 10-plex 128H | PSN-1 | 1000 | 0 |
| | TMT10: 10-plex 128L | PSN-1 | 0 | 0 |
| | TMT10: 10-plex 127H | Capan-1 | 1000 | 1000 |
| | TMT10: 10-plex 127L | Capan-1 | 1000 | 0 |
| | TMT10: 10-plex 126 | Capan-1 | 0 | 0 |

**Appendix Table S5. TMT-mapping for phospho-experiments in six additional PDAC cell lines (non-decryptM).** Each experiment was performed in quadruplicates. The channel containing AsPC-1 cells was used for inter-batch normalization.

| TMT Channel | Cell line | Time point | GEM or Elimusertib [nM] | Description |
|---|---|---|---|---|
| TMTpro: 16-plex 126 | AsPC-1 | 0 | 0 | Vehicle for 15 min |
| TMTpro: 16-plex 127N | AsPC-1 | 30 min | 1000 | - |
| TMTpro: 16-plex 127C | AsPC-1 | 30 min | 0 | Vehicle for 30 min |
| TMTpro: 16-plex 128N | AsPC-1 | 1 h | 1000 | - |
| TMTpro: 16-plex 128C | AsPC-1 | 1 h | 0 | Vehicle for 1 h |
| TMTpro: 16-plex 129N | AsPC-1 | 2 h | 1000 | - |
| TMTpro: 16-plex 129C | AsPC-1 | 2 h | 0 | Vehicle for 2 h |
| TMTpro: 16-plex 130N | AsPC-1 | 4 h | 1000 | - |
| TMTpro: 16-plex 130C | AsPC-1 | 4 h | 0 | Vehicle for 3 h |
| TMTpro: 16-plex 131N | AsPC-1 | 6 h | 1000 | - |
| TMTpro: 16-plex 131C | AsPC-1 | 6 h | 0 | Vehicle for 6 h |
| TMTpro: 16-plex 132N | AsPC-1 | 12 h | 1000 | - |
| TMTpro: 16-plex 132C | AsPC-1 | 12 h | 0 | Vehicle for 12 h |
| TMTpro: 16-plex 133N | AsPC-1 | 24 h | 1000 | - |
| TMTpro: 16-plex 133C | AsPC-1 | 24 h | 0 | Vehicle for 24 h |

**Appendix Table S6. TMT-mapping for time-dependent full proteome experiment in AsPC-1 cells.** Cells were either treated with 1,000 nM of GEM or with 1,000 nM Elimusertib (separate TMT experiments).

## References

Hornbeck PV, Zhang B, Murray B, Kornhauser JM, Latham V, Skrzypek E (2015) PhosphoSitePlus, 2014: mutations, PTMs and recalibrations. *Nucleic Acids Res* 43: D512-520

Thangudu RR, Rudnick PA, Holck M, Singhal D, MacCoss MJ, Edwards NJ, Ketchum KA, Kinsinger CR, Kim E, Basu A (2020) Abstract LB-242: Proteomic Data Commons: A resource for proteogenomic analysis. *Cancer Research* 80: LB-242-LB-242

Wang D, Qian X, Du YN, Sanchez-Solana B, Chen K, Kanigicherla M, Jenkins LM, Luo J, Eng S, Park B *et al* (2023) cProSite: A web based interactive platform for online proteomics, phosphoproteomics, and genomics data analysis. *J Biotechnol Biomed* 6: 573-578
