# Supplementary material for: Gemcitabine and ATR inhibitors synergize to kill PDAC cells by blocking DNA damage response
Source: Mol Syst Biol. 2025 Jan 21;21(3):231–53. doi: 10.1038/s44320-025-00085-6 (PMC11876601; doi:10.1038/s44320-025-00085-6)
Supplement: Supplementary file 20 — Expanded View Figures [file 44320_2025_85_MOESM20_ESM.pdf]

Expanded View Figures

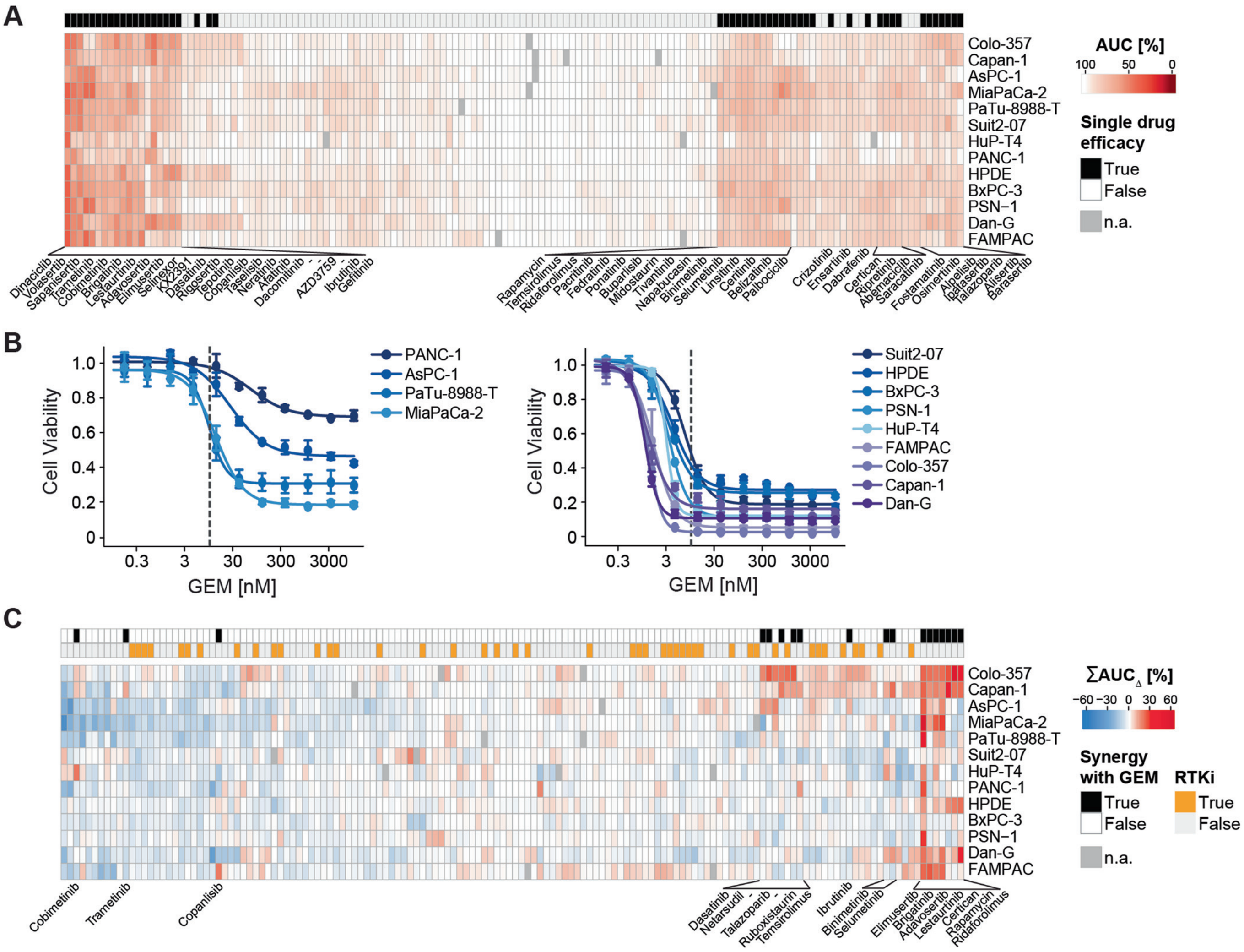

Figure EV1. Single drug and combination screening of 146 targeted inhibitors and GEM in 13 PDAC cell lines.

(**A**) Area-under-curve (AUC, in %) of single drug treatments for all 146 inhibitors across 13 PDAC cell lines. A lower AUC indicates greater efficacy. Drugs showing efficacy in at least one cell line are annotated in black, and labeled with text. Missing data (n.a.) is indicated in gray. (**B**) Cell viability upon treatment with increasing doses of GEM for all cell lines, relative to vehicle. Data are presented as mean values, with error bars representing the ± s.d. of duplicates ($n = 2$). Dashed line indicates an EC_{50} threshold of 10 nM, which was used to separate less sensitive cell lines (left) from more sensitive cell lines (right). (**C**) Summed shift in AUC (ΣAUC_{Δ}, in %) upon combination with GEM for all 146 library drugs across all cell lines. A higher ΣAUC_{Δ} indicates greater synergy. Drugs showing synergy in at least one cell line are annotated in black, and labeled with text. Inhibitors of receptor tyrosine kinases (RTKi) are annotated in orange. Missing data (n.a.) is indicated in gray.

© The Author(s)

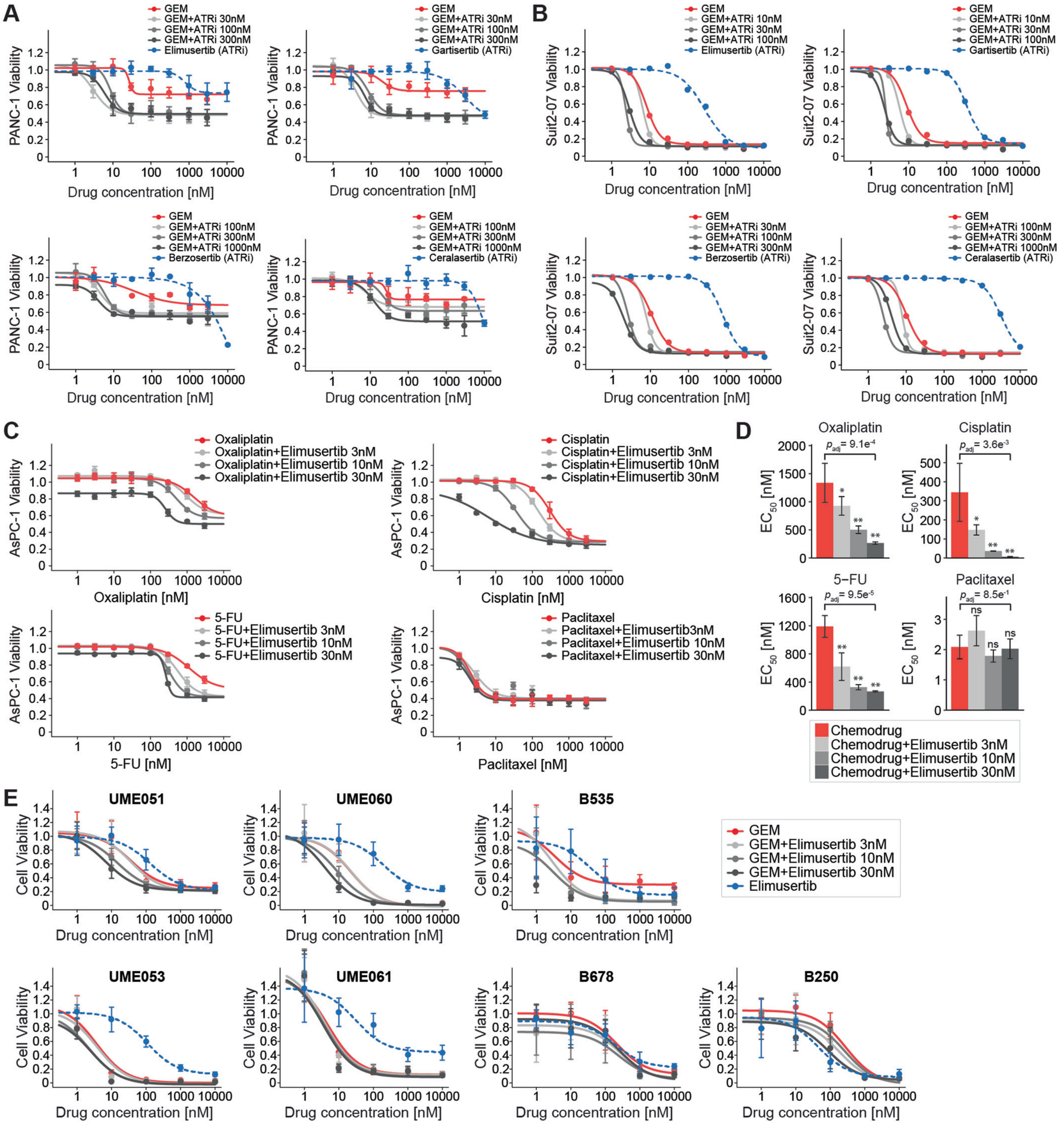

Figure EV2. Additional viability assays with ATRi in PDAC cell lines and PDOs.

(**A**, **B**) Cell viability of PANC-1 (**A**) and Suit2-07 (**B**) after treatment with GEM alone (red), GEM in combination with three sub-EC_{50} doses of ATR inhibitor (shades of gray), or ATR inhibitor alone (blue dotted) relative to vehicle. (**C**) AsPC-1 viability after treatment with four different chemodrugs alone (red) or chemodrugs in combination with three sub-EC_{50} doses of Elimusertib (shades of gray) relative to vehicle. (**D**) EC_{50} of four different chemodrugs alone (red) and chemodrugs in combination with three sub-EC_{50} doses of Elimusertib (shades of gray). Asterisks show the significance level from a Student's t-test against GEM monotherapy (*p*-values were adjusted using the Benjamini–Hochberg procedure; $*p_{adj} < 0.05$; $**p_{adj} < 0.01$; ns: non-significant). Adjusted *p*-values are shown only for the most significant combinations; for all others, refer to Dataset EV7. (**E**) Cell viability of seven PDOs treated with GEM alone (red), GEM in combination with three sub-EC_{50} doses of Elimusertib (shades of gray), or Elimusertib alone (blue dotted) relative to vehicle. Data information: Data are presented as mean values, with error bars representing the ± s.d. of triplicates (**A**, **C**, and **D**; $n = 3$) or eight replicates (**E**; $n = 8$). Data shown in (**B**) represent a single experiment ($n = 1$).

© The Author(s)

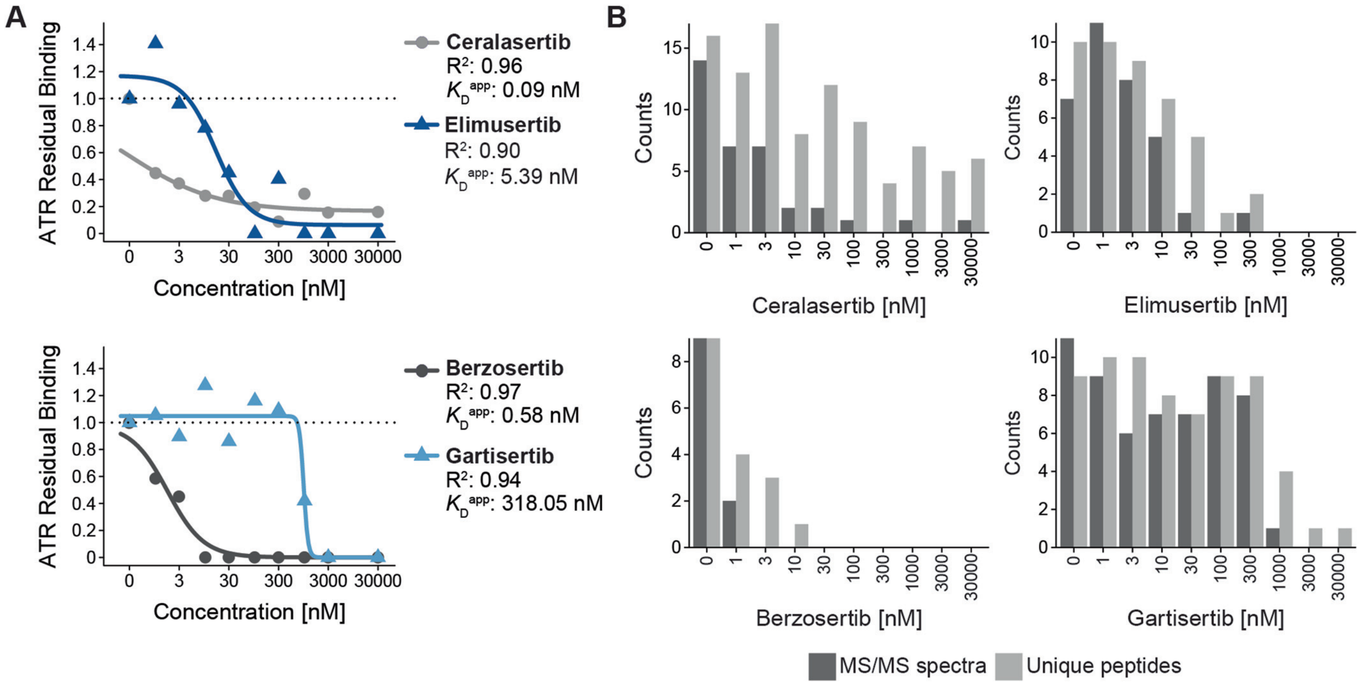

Figure EV3. Binding of ATR kinase by clinical inhibitors.

(**A**) Residual binding of ATR on Kinobeads upon increasing doses of Elimusertib and Ceralasertib (top), and Berzosertib and Gartisertib (bottom), based on label-free quantification (LFQ) intensities. Curve fit (R^2) and apparent affinity constants (K_D^{app}) are given in the legend. (**B**) Dose-dependent reduction in MS/MS spectra and unique peptides of ATR kinase in pulldown experiment with increasing concentrations of Elimusertib and Ceralasertib (top), and Berzosertib and Gartisertib (bottom).

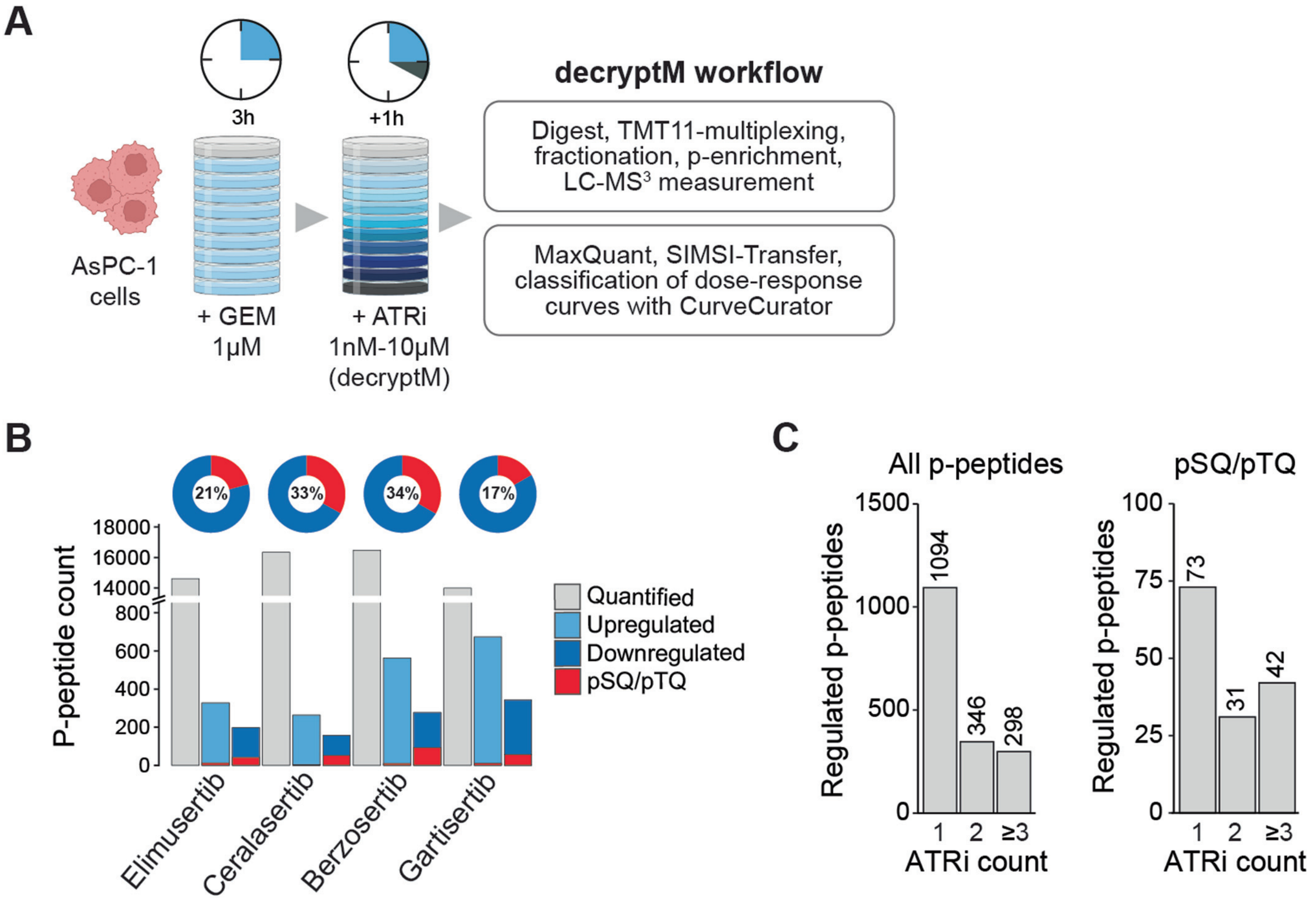

Figure EV4. Phosphoproteomic decryptM workflow to study ATR inhibition in DNA-damaged cells.

(**A**) Schematic workflow of decryptM experiments with four clinical ATR inhibitors in DNA-damaged AsPC-1 cells (pre-incubated with GEM). Seeded AsPC-1 cells were incubated with 1 µM GEM for 3 h, followed by nine doses of ATR inhibitor for one additional hour. TMT-labeled, fractionated and phospho-enriched peptides were measured by LC-MS^3. After data processing with MaxQuant and SIMSI-Transfer, dose–response data was analyzed using CurveCurator. (**B**) Number of quantified (gray), upregulated (light blue), or downregulated (dark blue) phosphorylated peptides in the four decryptM experiments. Peptides containing the pSQ/pTQ motif are highlighted in red, and the numbers in pie charts indicate the fraction of pSQ/pTQ motif-containing peptides within the down-regulated phosphoproteome. (**C**) Count of all p-peptides (left) or pSQ/pTQ motif-containing peptides (right) regulated by one, two, or at least three ATR inhibitors in decryptM experiments.

 © The Author(s)

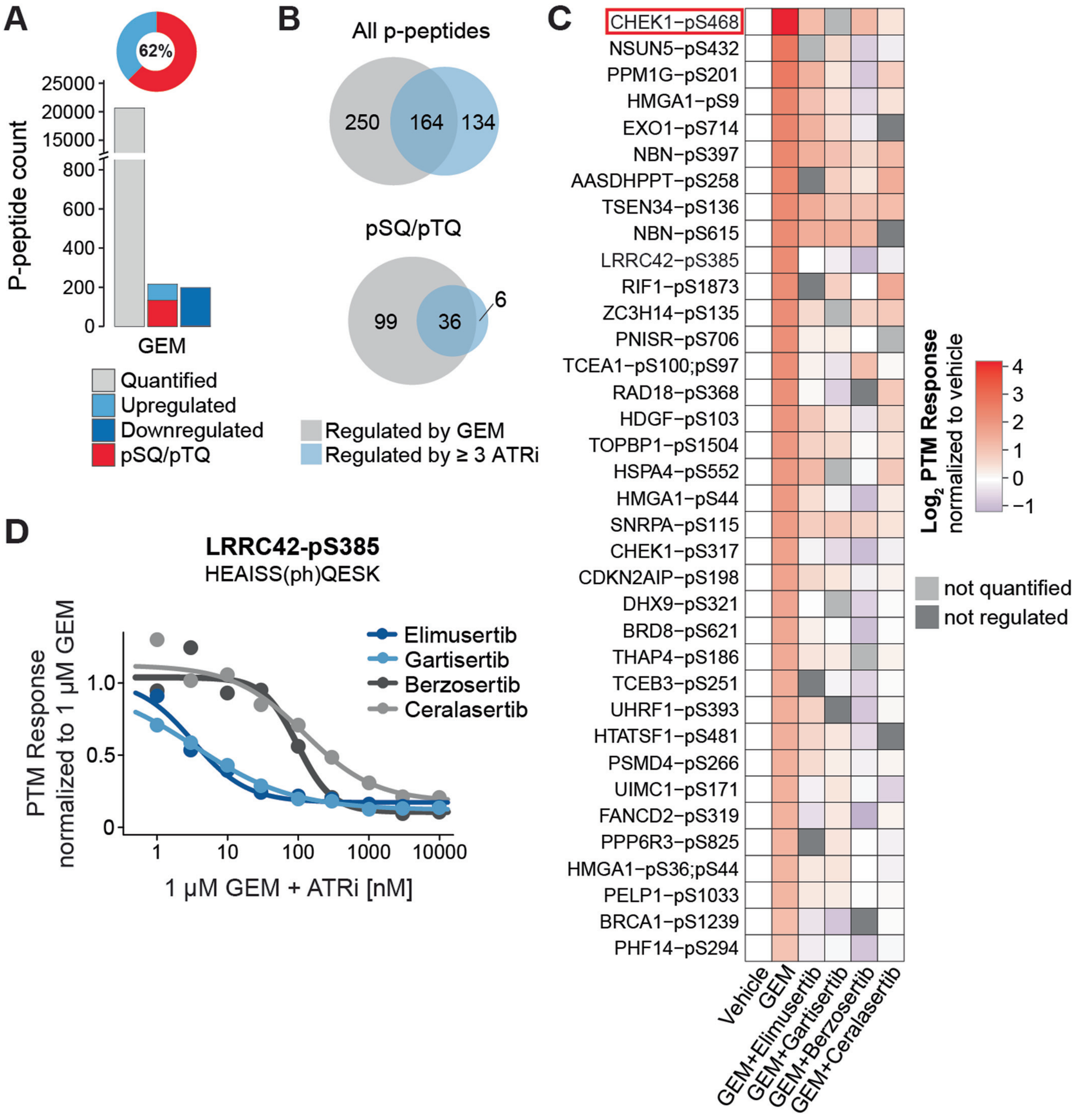

Figure EV5. 36 GEM-induced pSQ/pTQ sites are counter-regulated by ATRi.

(**A**) Barplot showing the number of all quantified (gray), upregulated (light blue), or downregulated (dark blue) phosphorylated peptides upon treatment of AsPC-1 cells with 1 µM GEM for 4 h ($n = 4$). Peptides containing the pSQ/pTQ motif are highlighted in red, and numbers in pie charts indicate the fraction of pSQ/pTQ peptides within the upregulated phosphoproteome. (**B**) Overlap in regulated phosphorylated peptides between GEM and at least three out of four ATR inhibitors. Left: all p-peptides, right: pSQ/pTQ motif-containing peptides. (**C**) Heatmap of phosphorylation sites induced by GEM and inhibited by at least three of the four ATR inhibitors. Log_2 fold changes in phosphorylation are shown relative to vehicle (no drug). Light gray indicates missing values (not quantified), and dark gray indicates quantified but not significantly regulated peptides. (**D**) Dose-dependent regulation of LRRC42-pS385 by the four ATR inhibitors in DNA-damaged cells. PTM response was normalized to 1 µM GEM.

© The Author(s)
